# Decreasing the time to achieve therapeutic vancomycin concentrations in critically ill patients: developing and testing of a dosing nomogram

**DOI:** 10.1186/s13054-014-0654-2

**Published:** 2014-12-05

**Authors:** João Pedro Baptista, Jason A Roberts, Eduardo Sousa, Ricardo Freitas, Nuno Deveza, Jorge Pimentel

**Affiliations:** Serviço de Medicina Intensiva, Centro Hospitalar e Universitário de Coimbra, Praceta Prof. Mota Pinto, 3000-075 Coimbra, Portugal; Burns, Trauma and Critical Care Research Centre, The University of Queensland, Brisbane, Queensland Australia; Department of Molecular and Clinical Pharmacology, University of Liverpool, Liverpool, UK; Department of Intensive Care Medicine, Royal Brisbane and Women’s Hospital, Brisbane, Queensland Australia; Pharmacy Department, Royal Brisbane and Women’s Hospital, Brisbane, Queensland Australia

## Abstract

**Introduction:**

Achievement of optimal vancomycin exposure is crucial to improve the management of patients with life-threatening infections caused by susceptible Gram-positive bacteria and is of particular concern in patients with augmented renal clearance (ARC). The aim of this study was to develop a dosing nomogram for the administration of vancomycin by continuous infusion for the first 24 hours of therapy based on the measured urinary creatinine clearance (8 h CL_CR_).

**Methods:**

This single-center study included all critically ill patients treated with vancomycin over a 13-month period (group 1), in which we retrospectively assessed the correlation between vancomycin clearance and 8 h CL_CR_. This data was used to develop a formula for optimised drug dosing. The efficiency of this formula was prospectively evaluated in a second cohort of 25 consecutive critically ill patients (group 2). Vancomycin serum concentrations between 20 to 30 mg/L were considered adequate. ARC was defined as 8 h CL_CR_ more than 130 ml/min/1.73 m^2^.

**Results:**

The incidence of ARC was 36% (*n* = 29/79) and 40% (10/25) in group 1 (*n* = 79) and 2 (*n* = 25), respectively. The mean serum vancomycin concentration on day 1 was 21.5 (6.4) and 24.5 (5.2) mg/L, for both groups respectively. On the treatment day, vancomycin plasma clearance was 5.12 (1.9) L/h in group 1 and correlated significantly with the 8 h CL_CR_ (r^2^ = 0.66; *P* <0.001). The achievement of adequate vancomycin serum concentrations in group 2 was 84% (*n* = 21/25) versus 51% (*n* = 40/79) – *P* <0.005.

**Conclusions:**

This new vancomycin nomogram enabled the achievement of adequate serum concentrations in 84% of the patients on the first day of treatment.

## Introduction

The emergence of multidrug-resistant bacteria has been associated with the inappropriate use and the inadequate dosing of antibiotics [1]. The EPIC II study showed that 51% of the patients admitted to ICU had infections and that 71% of all patients were receiving antibiotics [2]. In addition, this study demonstrates that infection by methicillin-resistant *Staphylococcus aureus* (MRSA) is particularly problematic. Compared to methicillin-susceptible *S. aureus* (MSSA), MRSA is independently associated with an almost 50% higher likelihood of hospital death [3] and has been considered as a serious threat by the Centers for Disease Control and Prevention (CDC) [4]. Despite its widespread use, vancomycin remains the first-line agent in the treatment of patients with MRSA infection, including those in the critical-care setting. In Portugal, MRSA prevalence is one of the highest in Europe and the emergence of vancomycin-resistant enterococci and vancomycin-resistant *S. aureus* is an area of particular concern [5,6].

Though there are limited data to support its routine use in patient care [7], the administration of vancomycin by continuous infusion (CI) has been used for the treatment of critically ill septic patients, because of its practical advantages: 1) rapid achievement of steady-state target concentrations; 2) lower variability in drug exposure; 3) simplicity of interpretation of therapeutic drug monitoring (TDM) and dose adjustment; 4) ease of administration; 5) lower rates of nephrotoxicity, 6) lower costs and 7) lower mortality [8-11]. However, achieving the desired serum concentration can still be difficult in this group of patients [11-14]. Amongst the several factors that contribute to the difficulties in establishing adequate dosing regimens [15], augmented renal clearance (ARC) is emerging as a new and crucial factor, since vancomycin is predominantly eliminated by the kidneys [16,17]. ARC refers to an enhanced elimination of circulating solute including drugs and is defined as a creatinine clearance exceeding 130 ml/minute. ARC appears to be quite common in sub-populations of critically ill patients and can lead to very low concentrations of renally cleared drugs like vancomycin.

The aim of this study was to develop a dosing nomogram for the administration of vancomycin by CI for the first 24 h of therapy based on an 8-h measured urinary creatinine clearance (8 h CL_CR_); and second, to evaluate its efficiency in a separate cohort of critically ill septic patients.

## Material and methods

### Study design

This single-center study was conducted in a 20-bed mixed ICU at the 1,375-bed Coimbra University Hospitals (Portugal). Data were collected retrospectively over a 13-month period from all consecutive, ventilated, adult patients with severe sepsis or septic shock who started empirical or directed treatment that included vancomycin (group 1). The intravenous treatment protocol started with a loading dose of vancomycin (Vancomicina Hikma®, Hikma Farmacêutica, Terrugem, Sintra, Portugal) based on the patient’s actual weight, of 1,000 mg (body weight ≤70 kg) or 1,500 mg (body weight >70 kg) over 1 to 2 h, followed by CI (30 mg/kg/day). Daily TDM (between 7:00 and 7:30 am) of serum vancomycin concentrations was performed, starting the next day (day 1). Serum concentrations between 20 and 30 mg/L were considered adequate [16]. An increased serum creatinine concentration >0.3 mg/dL on two or more consecutive days and so-called red-man syndrome were considered adverse effects related to vancomycin administration. Ototoxicity evaluation was not feasible during the study period. At Coimbra University Hospitals, *S. aureus* shows no resistance to vancomycin (minimum inhibitory concentration (MIC) ≤1 mg/L - European Committee on Antimicrobial Susceptibility Testing (EUCAST)).

Height, weight, body mass index (BMI) and body surface area (BSA) were measured. The DuBois and DuBois formula was used to calculate BSA as follows:

$$ \mathrm{B}\mathrm{S}\mathrm{A} = 0.007184 \times {\left(\mathrm{Height}\ \left(\mathrm{cm}\right)\right)}^{0.725} \times {\left(\mathrm{weight}\ \left(\mathrm{kg}\right)\right)}^{0.425}. $$

A daily 8 h CL_CR_ was collected during the patient’s ICU admission, between 23:00 h and 07:00 h, as part of the daily routine procedure in our unit. This measurement used a standard urinary collection (via indwelling catheter) for the 8-h period following measurement of creatinine concentration in urine (u) and blood (s) for calculation of 8 h CL_CR_ (ml/minute/1.73 m^2^), according to the formula:

1 $$ 8\;h\;C{L}_{CR}=\left(uCr/sCr\right)\times \left(8\;h\; urinary\; output/480\right)\times \left(1.73/BSA\right) $$

Augmented renal clearance was defined as 8 h CL_CR_ >130 ml/minute/1.73 m^2^. Exclusion criteria were the following: 1) need for renal replacement therapy; 2) serum creatinine concentration >1.3 mg/dL; 3) known chronic kidney disease; 4) age under 18 years; 5) pregnancy, and 6) ICU stay of less than 48 h. Using a previously described methodology [17,18], we calculated the vancomycin plasma clearance (CL_vanco_) according to the formula:

2 $$ C{L}_{vanco}\left(L/h\right)=IR\;\left( mg/h\right)\;/\;{C}_{ss}\left( mg/L\right) $$

Where IR represents infusion rate of vancomycin by CI and and *C*_*ss*_ represents the vancomycin serum concentration at pseudo steady-state. Relationship between CL_vanco_ and 8 h CL_CR_ was used to define a dosing nomogram for vancomycin for different 8 h CL_CR_ that targets a target C_ss_ of 25 mg/L. The rationale behind the choice of 25 mg/L as the ideal target was based on current recommendations and on the pharmacokinetic/pharmacodynamic (PK/PD) characteristics of vancomycin [16,19]. The resultant dosing nomogram was then prospectively applied for vancomycin dosing by CI in the ICU (after adequate loading dose). Thereafter, we collected data on the serum drug concentration on day 1 on the first 25 treated critically ill septic patients (group 2). Vancomycin treatment was initiated at the discretion of the ICU physician. Inclusion criteria for the second cohort were as following: 1) evaluation of 8 h CL_CR_ the day of initiation of vancomycin; 2) stable renal function; 3) administration of loading and maintenance dose per protocol, and 4) interval between loading dose and TDM for vancomycin >12 h and <24 h.

This study was approved by the Human Research Ethics Committee of Coimbra University Hospitals (CHUC-114-13), which waived the need for informed consent.

### Statistical analysis

The results were analyzed with the SPSS software package v.19.0 (SPSS Inc., Chicago, IL, USA) and with MedCalc software v.9.3.8 for Windows (MedCalc Software, Mariakerke, Belgium). Continuous variables are expressed as mean (standard deviation) or median (interquartile range) where applicable. Qualitative variables are presented as frequencies and percentages. Differences in categorical variables were calculated using Fisher’s exact test. For subgroup comparison of continuous data, the Student *t-*test was used. Linear regression was employed for curve fitting. A *P*-value of <0.05 was considered statistically significant.

## Results

**Figure 1:**
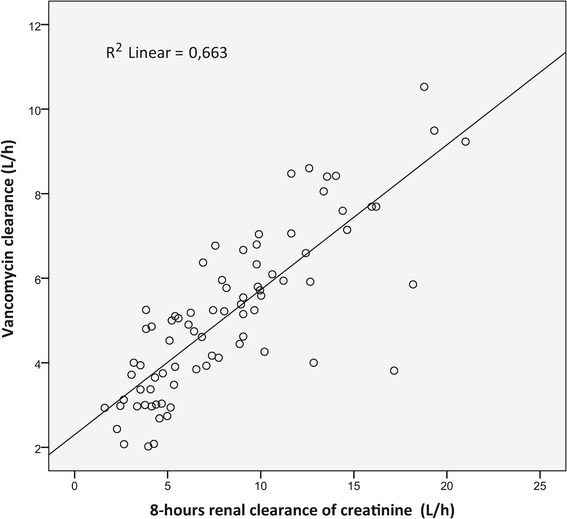
**Linear correlation between 8-hour measured urinary creatinine clearance and vancomycin clearance on day 1 in group 1 (79 patients).** *R* ^2^ = 0.663, *P* <0.001.

The main demographic characteristics of the patients belonging to groups 1 and 2 (79 and 25 patients, respectively) are shown in Table 1 and the dosing characteristics and observed pharmacokinetics of vancomycin treatment are described in Table 2. The predominant foci of the infection were lung (63.2%), skin and soft tissues (7.5%), bloodstream (6.3%) and abdominal (6.3%). Globally, the frequency of achievement of adequate vancomycin serum concentrations (20 to 30 mg/L) was 51% (n = 40/79) in group 1 versus 84% (n = 21/25) in group 2 (*P* <0.005). Of note, the population of group 1 showed a wide range of renal function, between 25 and 335 ml/minute/1.73 m^2^. The incidence of ARC in group 1 was 36% (n = 29/79) and the CL_vanco_ was 6.8 and 4.2 L/h in patients with and without ARC, respectively. Within these 29 patients showing ARC, only 28% achieved adequate vancomycin serum concentrations (20 to 30 mg/L); 74% of the remaining 50 patients achieved therapeutic concentrations. The 8 h CL_CR_ and CL_vanco_ on day 1 in group 1 was significantly linearly correlated (*r*^2^ = 0.66; *P* <0.001) (Figure 1).

**Table 1:** **Baseline characteristics of the studied population: group 1 (retrospective cohort) and group 2 (second cohort)**

**Demographics**	**Group 1**	**Group 2**	***p***
	**(79 patients)**	**(25 patients)**	
Male, number (%)	52 (66.0)	17 (68.0)	ns
Age, years	57.8 (15.5)	59.9 (17.2)	ns
Body weight, kg	77 (70-86)	75 (67.5-87.5)	ns
Body surface area, m^2^	1.87 (0.16)	1.86 (0.19)	ns
Body mass index, kg/m^2^	28.1 (25.3-30.4)	25.7 (24.4-30.6)	ns
New simplified acute physiology score	39 (34-50)	43 (37-46)	ns
8 h CL_CR_ on day 1, mL/min/1.73 m^2^	125.1 (66.5)	120.5 (54.2)	ns
Baseline serum creatinine, mg/mL	0.68 (0.30)	0.68 (0.31)	ns
Lowest serum creatinine, mg/mL*	0.57 (0.20)	0.60 (0.19)	ns
Highest serum creatinine, mg/mL*	0.73 (0.28)	0.81 (0.45)	ns
Patients with serum creatinine increase >0.3 mg/mL, number (%)*	5 (6.3)	1 (4.0)	ns
Patients with ARC on day 1, number (%)	29 (36.7)	10 (40.0)	ns
Mechanical ventilation on day 1, number (%)	79 (100)	25 (100)	ns
Admission days	19 (9-29)	23 (18-30)	ns
**Admission group diagnosis, %**			
Trauma admission	44.3	52.0	ns
Surgical admission	16.5	28.0	ns
Medical admission	39.2	20.0	ns

Quantitative variables were expressed as mean (standard deviation) or median (interquartile range) when applied. *During the vancomycin treatment. Augmented renal clearance (ARC) defined as 8 h CL_CR_ >130 mL/min/1.73 m^2^; 8 h CL_CR_, 8-hour measured urinary creatinine clearance; ns, non significant.

**Table 2:** **Dosing information and pharmacokinetics of vancomycin in the retrospective cohort (group 1) and in the second cohort (group 2)**

	**Group 1**	**Group 2**
	**(79 patients)**	**(25 patients)**
Loading dose of vancomycin on day 0, mg	1000 (1000-1500)	1500 (1000-1500)
Loading dose of vancomycin on day 0, mg/kg	14.3 (12.8-17.6)	18.8 (16.7-21.4)
Perfusion dose of vancomycin on day 0, mg	1920 (1512-2400)	2072 (1750-2622)
Total dosing of vancomycin on day 0, mg	3160 (2520-3880)	3584 (2976-4138)
Time interval (h) between vancomycin perfusion and TDM	18 (17-27)	18 (17-19)
Serum vancomycin concentration on day 1, mg/L	20.6 (16.7-26)	24.5 (22.2-27.4)
Clearance of vancomycin on day 1, L/h	5.1 (1.9)	NC

Values are expressed as median and interquartile range [Q25-Q75] except for Clearance of vancomycin on day 1 (mean and standard deviation).

TDM, therapeutic drug monitoring; day 0, the day before day 1, corresponding to the day of the administration of vancomycin; NC, not calculated.

The equation from the linear regression was as follows:

3 $$ C{L}_{vanco}\left(L/h\right)=0.021\;x\;8\;h\;C{L}_{CR}\left( mL/ min\right)+2.3 $$

Using equation 3, a new equation was developed for calculating a continuous infusion vancomycin dose per day, considering 25 mg/L as the preferred target:

4 $$ \begin{array}{c}\hfill Vancomycin\; dose\;\left(g/d\right)=\left(0.021\;x\;8\;h\;C{L}_{CR}+2.3\right)\times 25\left( mg/L\right)\times 24/1000\;\hfill \\ {}\hfill =\left(0.021\;x\;8\;h\;C{L}_{CR}+2.3\right)\times 0.6\hfill \end{array} $$

**Figure 2:**
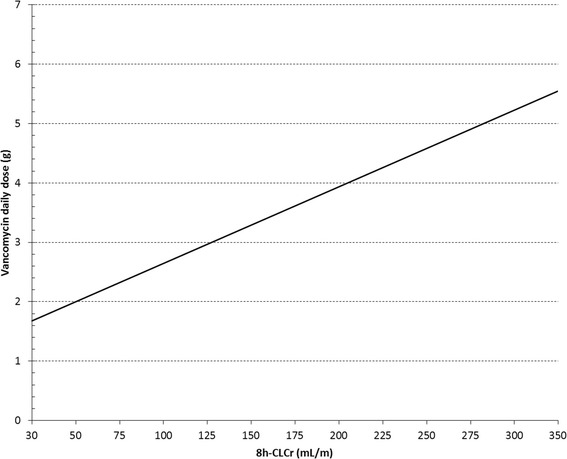
**Nomogram for calculation of the daily vancomycin dosage (g/24 h) administered by continuous infusion required for achievement of target drug concentration (25 mg/L) based on 8-hour measured urinary creatinine clearance.** 8 h CL_CR_ = 8-hour measured urinary creatinine clearance.

**Figure 3:**
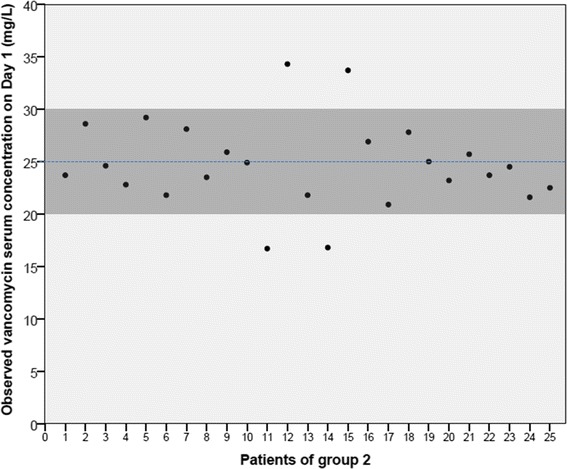
**Distribution of the serum vancomycin concentrations within the 25 patients belonging to group 2.** The grey area represents the target interval for serum vancomycin on the day 1 of treatment (serum concentrations between 20 and 30 mg/L).

We then used equation 4 to develop a dosing nomogram for vancomycin dosing in the first 24 h, after a loading dose (Figure 2). As a result of the application of this nomogram on group 2 (n = 25 patients), we observed that 21 (84%) achieved serum concentrations between 20 and 30 mg/L on day 1. Two patients (8%) exceeded these limits (34.3 and 33.7 mg/L) and another two patients did not meet the target interval (16.8 and 16.7 mg/L). Of note, all the patients with ARC belonging to group 2 were in the target concentration range (n = 10/10), meaning that all the under- and over-treated patients were non-ARC patients (4/15, 26.6%). The observed vancomycin serum concentrations within the 25 patients, and the respective visual interrelation with the target interval (20 to 30 mg/L) and preferred target concentration (25 mg/L) is showed in the Figure 3. With the exception of one patient who had an increase of over 0.3 mg/dL of serum creatinine concentration in two consecutive days without needing treatment interruption (1/25, 4.0%), no clinical or laboratory vancomycin-related side effects were noted during the period of treatment at the ICU within group 2. On the other hand, the incidence of nephrotoxicity during vancomycin treatment in group 1 was 6.3% (5/79) (Table 1).

## Discussion

Our results show that in a broad population of adult ICU patients treated with continuously infused vancomycin, the use of a dosing nomogram significantly increased the achievement of therapeutic concentrations in the first 24 h of treatment, particularly in the patients exhibiting ARC. Application of this nomogram was found to be easy, user-friendly and effective. The nomogram requires the availability of an 8 h CL_CR_ and vancomycin serum concentration monitoring, which is not available in all ICUs, but these results demonstrate how beneficial such tests can be to ensure more accurate antibiotic dosing.

Vancomycin is a glycopeptide antibacterial agent that is predominantly excreted unchanged in urine by glomerular filtration and as such, kidney function is a determinant factor of vancomycin pharmacokinetics and dosing. The best measure of kidney function is the glomerular filtration rate (GFR). Urinary creatinine clearance seems to be the best clinical surrogate of renal function, taking into account its applicability at the bedside, its reliability and the negligible costs associated, provided that clinicians are aware of its limitations [20-22]. Estimates of GFR using equations based on serum creatinine concentrations are flawed in the critically ill patient [21,23]. Taking all of this information together, the measurement of the renal clearance of creatinine in this setting could be used, from a clinical point of view, as the single most accessible parameter allowing appraisal of pharmacokinetic characteristics of the critically ill patient.

Clinicians are used to adjusting the dosing of antibiotics according to acute or chronic renal failure, however the adjustment to elevated function of the kidneys is considered to be quite rare, and there are no recommendations on this clinical issue. Moreover, the incidence of ARC (here defined as CL_CR_ >130 ml/minute/1.73 m^2^) is probably high and ubiquitous in every ICU with an underestimated incidence [24]. Indeed ARC is being increasingly described, with previously reported rates, varying widely between 18% to as high as 57% in critically ill patients without renal dysfunction [23,24].

In the context of severe infection, a major consequence of ARC is the high renal clearance of hydrophilic antibiotics leading to a risk of inducing sub-therapeutic concentrations, therapeutic failure, emergence of multi-resistant bacterial strains, and even potentially increased mortality [25]. To date, different antibiotics have been studied in the presence of ARC and these reports have shown a strong association between ARC and sub-therapeutic concentrations [12,26,27]. Importantly for vancomycin, low serum concentrations are associated with decreases in susceptibility and in treatment failure of patients with MRSA infections [28]. A recent large-scale multicentre point-prevalence study revealed that a substantial proportion of critically ill patients treated with vancomycin did not achieve the target vancomycin concentration and showed high variability in pharmacokinetics parameters, supporting a re-evaluation of vancomycin dosing recommendations in this particular setting [29]. Furthermore, a recent consensus review recommended more aggressive vancomycin dosing to ensure achievement of the pharmacodynamic index associated with efficacy [16]. Though vancomycin is widely used in the ICU, there are relatively few studies focused on the early optimization of serum concentrations where CI is the prescribed mode of administration [18,30-35]. Surprisingly, only three studies among these used measured renal clearance of creatinine [32-34], three used mathematical estimates of renal function [18,31,35] and one study did not provide this information [30]. Among those studies evaluating the serum vancomycin concentration on day 1 or day 2, the achievement of target concentrations of 20 to 30 mg/L ranged between 48 and 52% [30,32].

A recent study described a new regimen for CI of vancomycin during continuous renal replacement therapy, which allowed the achievement of target drug concentrations in 63% of patients at 24 h [36]. Our study, using a dosing regimen guided by a dosing nomogram that is based on the 8 h CL_CR_ and after an adequate loading dose, permitted us to reach the target drug concentration in most patients on day 1 (n = 21/25, 84%), providing optimal and early antibiotic exposure in the septic patient, with negligible secondary effects (only one patient with a minor increase in serum creatinine concentration, without evolution to renal failure or need for interruption of the treatment). Roberts *et al.* conducted a population pharmacokinetic analysis of vancomycin CI in a large cohort of critically ill patients, using a Monte Carlo dose simulation for different total body weight, for different creatinine clearances and for different weight-based dosing vancomycin CI regimens [35]. The authors found that higher-than-recommended loading and daily doses of vancomycin seem to be necessary to rapidly achieve therapeutic serum concentrations in these patients. In addition, they state that a patient with a CL_CR_ of 100 ml/minute/1.73 m^2^ would require at least 35 mg/kg per day by CI to maintain target concentrations. Curiously, when we use the average weight of our 25 patients belonging to group 2 (75 kg), we found very similar results: 2.625 mg versus 2.600 mg (according to Table 3) in a period of 24 h, respectively. Both approaches seem to exhibit some complementarity: a retrospective development of a model and, on the other hand, a clinical prospective validation of a nomogram, respectively.

**Table 3:** **Daily vancomycin dosage administered by continuous infusion for achievement of target drug concentration (25 mg/L) for different values of creatinine clearance using two different nomograms**

**Creatinine clearance, mL/minute**	**Vancomycin dosing, g/24 h**	
	**Baptista** ***et al*** **. (present study)**	**Pea** ***et al*** **. [** 18 **]**
30	1.8	1.1
50	2.0	1.4
75	2.3	1.9
100	2.6	2.3
125	3.0	2.7
150	3.3	3.2
175	3.6	3.6
200	3.9	4.0
225	4.2	4.5
250	4.5	4.9
275	4.8	5.3
300	5.2	5.8
350	5.8	6.7

To the best of our knowledge, the study by Pea *et al.* is the only to provide and validate two user-friendly dosing nomograms for the treatment of critical ill patients with vancomycin by CI [18]. They described a significant correlation between CL_vanco_ and creatinine clearance (*r*^2^ = 0.56, *P* <0.001) and between the observed and the predicted serum drug concentration (*r*^2^ = 0.64, *P* <0.001), confirming the dependency of vancomycin elimination on the renal function. However, as acknowledged by the authors, the renal performance was evaluated by the Cockroft-Gault formula, which is a limitation of the study [21,23]. Of interest and despite this, when we created a new dosing nomogram based on the formula described by these authors, but using the same ideal target that we used in our study (25 mg/L), we obtained similar results for the calculation of the daily vancomycin dosage by CI to achieve target drug concentration (Table 3), confirming in two independent studies the need for a higher dosage to achieve adequate concentrations in the first 24 h of treatment with vancomycin by CI. The similar methodology applied in both studies, similar populations, exclusion of patients with prolonged ICU admission, and choice of the Cockroft-Gault formula by Pea and coworkers (showing higher accuracy when compared with other estimates of renal creatinine clearance [23]) may be possible explanations for the similarities between the two nomograms. On the other hand, the lower incidence of ARC in both cohorts (approximately 15%) when compared to those in our study (36.7 and 40.0% in group 1 and 2, respectively) and the recent literature describing lower accuracy of Cockroft-Gault estimates in both extremes of the normal range of 8 h CL_CR_ [23,37] may be possible explanations for the lower agreement between the two nomograms, particularly when considering low and very high values of 8 h CL_CR_.

Altogether, our study shows that it is possible to increase the likelihood of target attainment in the first 24 h of treatment with vancomycin, with potential benefits including better outcome and reduction of the development of bacterial resistance. Our study strengths lie in the considerable number of patients included in the retrospective cohort, the significant correlation obtained between measured 8 h CL_CR_ and CL_vanco_ (*r*^2^ = 0.66, *P* <0.001), and the wide range of 8 h CL_CR_ exhibited by these patients (25 to 335 ml/minute/1.73 m^2^). Therefore, this study is based on a representative cohort of septic critically ill patients, making it applicable in various levels of renal function, including patients with ARC - a sub-group with particular risk of under-treatment with vancomycin [12].

However, some limitations should be acknowledged. First, this was a single-center study, and therefore an extrapolation of the findings to other settings must be done with caution. Second, the second cohort (group 2) was smaller, giving less certainty to our conclusions. Third, 8 h CL_CR_ cannot be accepted as a gold-standard method to assess kidney function: its determination requires creatinine concentrations to be at steady-state, which is a rarely reached condition in critically ill patients. Finally, pharmacokinetic studies require a constant physiological status, leading us again to the absence of physiological stability in the critical-care setting. In addition, it is possible that not all patients were at actual steady-state, given that 11% of patients in group 1 had an interval between commencement of vancomycin infusion and sampling for TDM less than 16 h.

## Conclusions

A novel and easy-to-use vancomycin dosing nomogram for the first 24 h of treatment, based on the 8-h renal clearance of creatinine has been developed and prospectively shown to be effective in septic and critically ill patients at a teaching hospital.

## Key messages

- Augmented renal clearance appears to be quite common in sub-populations of critically ill patients and can lead to very low serum concentrations of vancomycin on the first day of treatment
- Clinicians are used to adjusting the dosing of antibiotics according to renal failure; however the adjustment to elevated function of the kidneys appears important to ensure target concentrations are achieved
- This study prospectively validated a new vancomycin dosing nomogram based on the 8-hours renal clearance of creatinine and demonstrated that it is possible to increase the likelihood of target attainment in the first 24 h of treatment, particularly in patients with augmented renal clearance
- Adequate serum concentrations of vancomycin should be confirmed with therapeutic drug monitoring, particularly in patients with extreme renal function alteration

## Abbreviations

8 h CL_CR_: 8-hour measured urinary creatinine clearance
APACHE II: Acute physiology and chronic health evaluation II score
ARC: augmented renal clearance
B: blood
BMI: body mass index
BSA: body surface area
CDC: Centers for Disease Control and Prevention
CI: continuous infusion
CL_vanco_: vancomycin plasma clearance
*C*_*ss*_: vancomycin serum concentration at pseudo steady-state
EUCAST: European Committee on Antimicrobial Susceptibility Testing
GFR: glomerular filtration rate
IR: infusion rate of vancomycin by continuous infusion
MIC: minimum inhibitory concentration of the suspected bacteria
MRSA: methicillin-resistant *Staphylococcus aureus*
MSSA: methicillin-susceptible *Staphylococcus aureus*
PD: pharmacodynamics
PK: pharmacokinetics
sCr: serum creatinine
TDM: therapeutic drug monitoring
u: urine
